# Adherence of Clinical Practice Guidelines for Pharmacologic Treatments of Hospitalized Patients With COVID-19 to Trustworthy Standards: A Systematic Review

**DOI:** 10.1001/jamanetworkopen.2021.36263

**Published:** 2021-12-10

**Authors:** Karen E. A. Burns, Matthew Laird, James Stevenson, Kimia Honarmand, David Granton, Michelle E. Kho, Deborah Cook, Jan O. Friedrich, Maureen O. Meade, Mark Duffett, Dipayan Chaudhuri, Kuan Liu, Frederick D’Aragon, Arnav Agarwal, Neill K. J. Adhikari, Hayle Noh, Bram Rochwerg

**Affiliations:** 1 Interdepartmental Division of Critical Care Medicine, Department of Medicine, Temerty Faculty of Medicine, University of Toronto, Toronto, Ontario, Canada; 2 Departments of Critical Care and Medicine, Unity Health Toronto, St Michael’s Hospital, Toronto, Ontario, Canada; 3 Departments of Medicine, Critical Care Medicine, Pediatrics and Health Research Methods, Evidence, and Impact, McMaster University, Hamilton, Ontario, Canada; 4 Li Ka Shing Knowledge Institute, St Michael’s Hospital, Toronto, Ontario, Canada; 5 School of Medicine, Royal College of Surgeons, Dublin, Ireland; 6 Department of Critical Care Medicine, London Health Sciences Centre, London, Ontario, Canada; 7 Department of Medicine, Western University, London, Ontario, Canada; 8 Physiotherapy and Division of Critical Care, St Joseph’s Healthcare, Hamilton, Ontario, Canada; 9 School of Rehabilitation Science, Faculty of Health Science, McMaster University, Hamilton, Ontario, Canada; 10 Hamilton Health Sciences, Hamilton, Ontario, Canada; 11 Dalla Lana School of Public Health and the Institute of Health Policy, Management, and Evaluation, University of Toronto, Toronto, Ontario, Canada; 12 Canadian Donation and Transplant Research Program, Ottawa, Ontario, Canada; 13 Department of Anesthesiology, Université de Sherbrooke, Sherbrooke, Quebec, Canada; 14 Department of Critical Care Medicine, Sunnybrook Health Sciences Centre, Toronto, Ontario, Canada

## Abstract

**Importance:**

The COVID-19 pandemic created the need for rapid and urgent guidance for clinicians to manage COVID-19 among patients and prevent transmission.

**Objective:**

To appraise the quality of clinical practice guidelines (CPGs) using the National Academy of Medicine (NAM) criteria.

**Evidence Review:**

A search of MEDLINE, EMBASE, and the Cochrane Central Register of Controlled Trials to December 14, 2020, and a search of related articles to February 28, 2021, that included CPGs developed by societies or by government or nongovernment organizations that reported pharmacologic treatments of hospitalized patients with COVID-19. Teams of 2 reviewers independently abstracted data and assessed CPG quality using the 15-item National Guideline Clearinghouse Extent of Adherence to Trustworthy Standards (NEATS) instrument.

**Findings:**

Thirty-two CPGs were included in the review. Of these, 25 (78.1%) were developed by professional societies and emanated from a single World Health Organization (WHO) region. Overall, the CPGs were of low quality. Only 7 CPGs (21.9%) reported funding sources, and 12 (37.5%) reported conflicts of interest. Only 5 CPGs (15.6%) included a methodologist, described a search strategy or study selection process, or synthesized the evidence. Although 14 CPGs (43.8%) made recommendations or suggestions for or against treatments, they infrequently rated confidence in the quality of the evidence (6 of 32 [18.8%]), described potential benefits and harms (6 of 32 [18.8%]), or graded the strength of the recommendations (5 of 32 [15.6%]). External review, patient or public perspectives, or a process for updating were rare. High-quality CPGs included a methodologist and multidisciplinary collaborations involving investigators from 2 or more WHO regions.

**Conclusions and Relevance:**

In this review, few COVID-19 CPGs met NAM standards for trustworthy guidelines. Approaches that prioritize engagement of a methodologist and multidisciplinary collaborators from at least 2 WHO regions may lead to the production of fewer, high-quality CPGs that are poised for updates as new evidence emerges.

**Trial Registration:**

PROSPERO Identifier: CRD42021245239

## Key Points

**Question:**

Do clinical practice guidelines (CPGs) that report on pharmacologic treatments of hospitalized patients with COVID-19 meet the National Academy of Medicine standards for trustworthiness?

**Findings:**

In this systematic review of 32 CPGs of predominantly low quality, few reported funding sources or conflicts of interest, included a methodologist, described a search strategy or study selection process, or synthesized evidence. Although 14 CPGs (43.8%) made recommendations or suggestions for or against treatments, they infrequently rated the confidence in the quality of the evidence (6 [18.8%]), described potential benefits and harms (6 [18.8%]), or graded the strength of recommendations (5 [15.6%]).

**Meaning:**

The findings of this study suggest that few COVID-19 CPGs meet National Academy of Medicine standards for trustworthy guidelines.

## Teaser

This systematic review evaluates the quality and trustworthiness of clinical practice guidelines for pharmacologic treatment of hospitalized patients with COVID-19 using the National Guideline Clearinghouse Extent of Adherence to Trustworthy Standards instrument.

## Introduction

Clinical practice guidelines (CPGs) should be systematically developed statements and recommendations that articulate the roles for diagnostic tests and treatments to inform clinician and patient decisions. The process for creating guidelines affects CPG quality. In turn, CPG quality affects patient care, safety, and health care equality. In 2011, the National Academy of Medicine (NAM) (formerly known as the Institute of Medicine)^1^ published a report stipulating that CPG recommendations should be supported by a systematic review of the evidence and highlighted 8 criteria for assessing the trustworthiness of CPGs.

Many instruments and scorecards have been developed to evaluate CPG quality.^2,3,4,5,6,7,8,9,10,11^ The Appraisal of Guidelines for Research and Evaluation (AGREE) II tool^8^ is the most widely used CPG appraisal tool. The scope of the AGREE II tool targets all components of a CPG report, emphasizing features that enhance its internal validity. The AGREE Recommendation Excellence tool,^9^ a supplement to the AGREE II tool, highlights 9 items in 3 themes that focus on the quality of the CPG recommendations and the justifications that underpin them. The AGREE Recommendation Excellence tool ascertains whether CPGs are credible and implementable by assessing their internal consistency. Notwithstanding, the AGREE II and AGREE Recommendation Excellence appraisal tools do not directly address the NAM criteria for trustworthy CPGs or consider the perspectives of different stakeholder groups involved in CPG development. More recently, the US Agency for Healthcare Research and Quality developed the National Guideline Clearinghouse Extent of Adherence to Trustworthy Standards (NEATS) instrument ^11^ (eMethods in Supplement 1) to provide a standardized approach to assess CPG quality. The NEATS tool explicitly evaluates the NAM criteria and assesses CPGs from a broad and multidisciplinary perspective.

The COVID-19 pandemic created the need for rapid and urgent guidance for clinicians to manage COVID-19 among patients and prevent transmission, but methodological rigor has been variable across CPGs.^12^ We systematically reviewed published CPGs reporting pharmacologic treatments for hospitalized patients with COVID-19 and evaluated their quality and trustworthiness using the NEATS instrument. We hypothesized that CPGs created and disseminated during the pandemic have important methodological weaknesses that affect their quality and trustworthiness.

## Methods

### Study Design

We systematically reviewed published CPGs addressing pharmacologic treatments for hospitalized patients with COVID-19. An ethics review was not obtained for this secondary analysis of published data.

### Data Sources and Searches

We searched MEDLINE, EMBASE, and the Cochrane Central Register of Controlled Trials (to December 14, 2020) and conducted a search of related articles (to February 28, 2021) to identify updates. Two reviewers (M.L. and J.S.) independently screened all titles and abstracts of citations for eligibility. Disagreements were resolved by consensus or in discussion with a third reviewer (K.E.A.B.).

### Study Selection

Eligible CPGs were investigator led, sponsored or produced by a national or international scientific organization or government or nongovernment organization related to global health, and reported on pharmacologic treatments of hospitalized patients with COVID-19 and its complications. Pharmacologic interventions referred to treatments dispensed by hospital pharmacies, with an identifiable molecular structure. We included all versions of published CPGs. We did not apply language restrictions. We excluded CPGs produced for regional or local use (eg, hospital-based), nonpharmacologic interventions, medications not dispensed by a hospital pharmacy (eg, herbal remedies, homeopathic medications), and CPGs that focused on treatments for specific populations (ie, obstetrical populations hospitalized with COVID-19).

### Data Abstraction

Fifteen reviewers (K.E.A.B., M.L, J.S., K.H., D.G., M.E.K., D.C., J.O.F., M.O.M., M.D., D.C., F.D., A.A., N.K.J.A., and B.R.), working independently and in duplicate, abstracted data pertaining to CPG publications (dates of submission, acceptance, and publication online and in print), geographical representation of collaborators using World Health Organization (WHO) regions, CPG sponsorship (professional society, government or nongovernment agency, or other), funding (monetary or nonmonetary), and scope (international, national, state/province, or other). Reviewers recorded the patient populations addressed (hospitalized, ward, intensive care unit, or other) and assessed whether CPGs had a formal conflict of interest (COI) policy and declared COIs (financial, nonfinancial, or both). They noted whether patient and/or public perspectives were sought or incorporated. For each pharmacologic intervention, appraisers documented outcomes of interest and the direction of the recommendation statement (for or against or no recommendation), the strength of the recommendation, and the certainty of the evidence.

### Quality Assessment

Working in pairs, reviewers appraised the quality of included CPGs using the NEATS instrument^11^ (eMethods in Supplement 1). Disagreements were resolved by consensus or in discussion with a third reviewer (K.E.A.B.).

### Data Synthesis and Analysis

We collated data in Excel, version 2016 (Microsoft Corporation), to characterize CPGs, NEATS scores, and the direction of recommendation or suggestion statements (for, against, or no recommendation) for each pharmacologic treatment described in included CPGs. The NEATS instrument includes 3 binary or categorical items reflecting adherence to the NAM standard: assessing disclosure of funding (yes or no), multidisciplinary representation (yes, no, or unknown), and inclusion of a methodologist on the guideline panel (yes, no, or unknown) in CPG panels and 12 Likert scales. Reviewers rated adherence (on a scale ranging from 1 [low adherence] to 5 [high adherence]) to 12 NAM standards reflecting disclosure/management of COIs, inclusion of patient and public perspectives, use of systematic review of the evidence (separate items for reporting search strategy, study selection, and synthesis of the evidence), a process for making recommendations (separate items for reporting grading or rating of the quality or strength of evidence, reporting benefits and harms of recommendations, including evidence summary supporting recommendations, and rating the strength of recommendations), generation of specific and unambiguous recommendations, procurement of external reviews, and inclusion of a prespecified process to update the CPG.

We tabulated results to highlight the evolution of evidence over time for each pharmacologic intervention addressed by 3 or more CPGs. We characterized and compared recommendations for use of each specific pharmacologic intervention by assessing whether a recommendation or suggestion was made for or against its use or whether no recommendation for its use was made. In assessing the consistency between CPGs for each pharmacologic treatment, we prioritized the direction of the recommendation over the strength of the recommendation.

**Figure 1.**
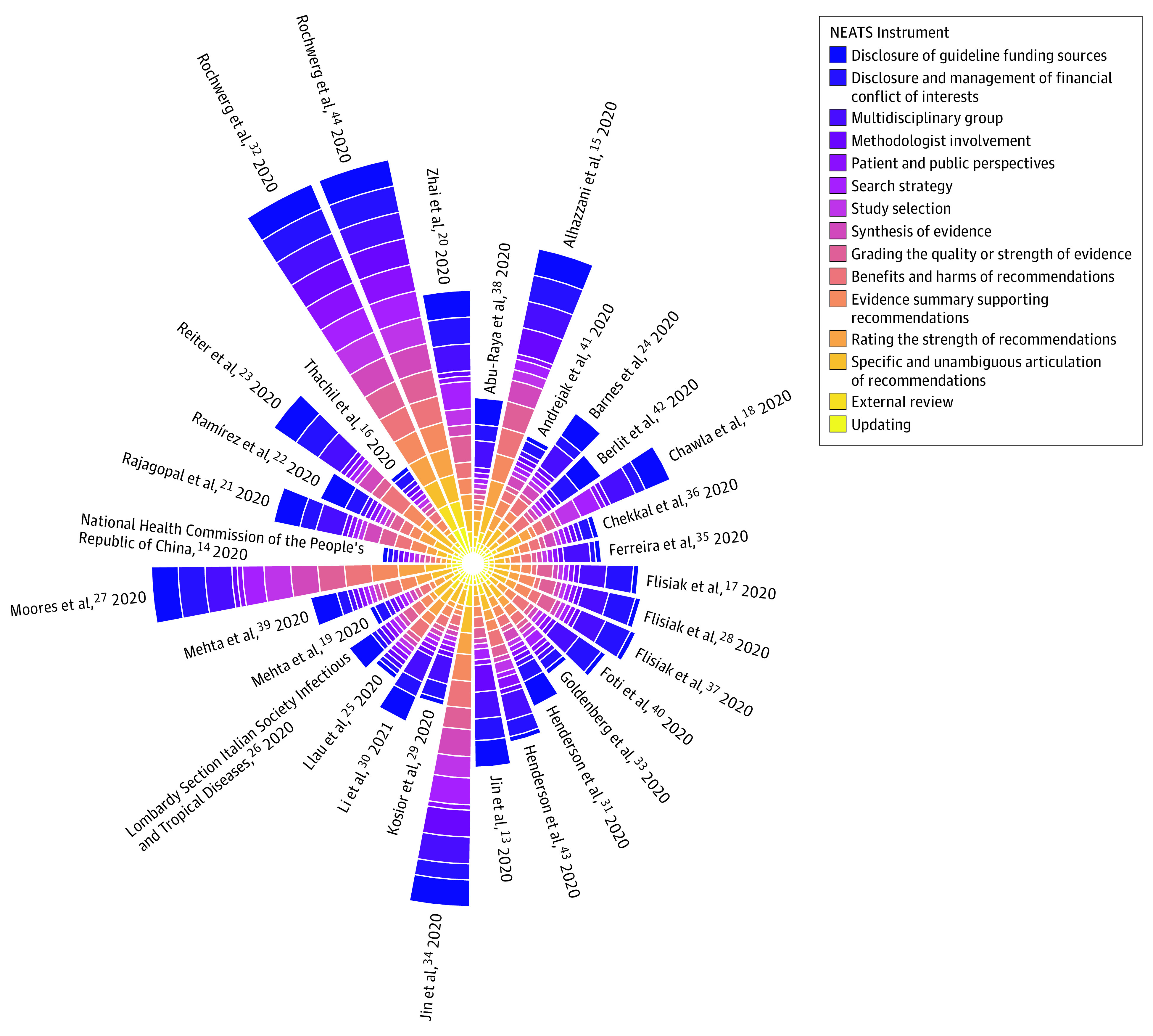
Coxscomb Chart Depicting the National Guideline Clearinghouse Extent of Adherence to Trustworthy Standards (NEATS) Score of Included Clinical Practice Guidelines Different colors reflect the various components of the National Academy of Medicine (NAM) score. Higher rays represent higher-quality scores as assessed using the NEATS instrument, which measures adherence (on a scale ranging from 1 [low adherence] to 5 [high adherence]) to 12 NAM standards and includes 3 binary or categorical questions (response options yes, no, and/or unknown).^11^

**Figure 2.**
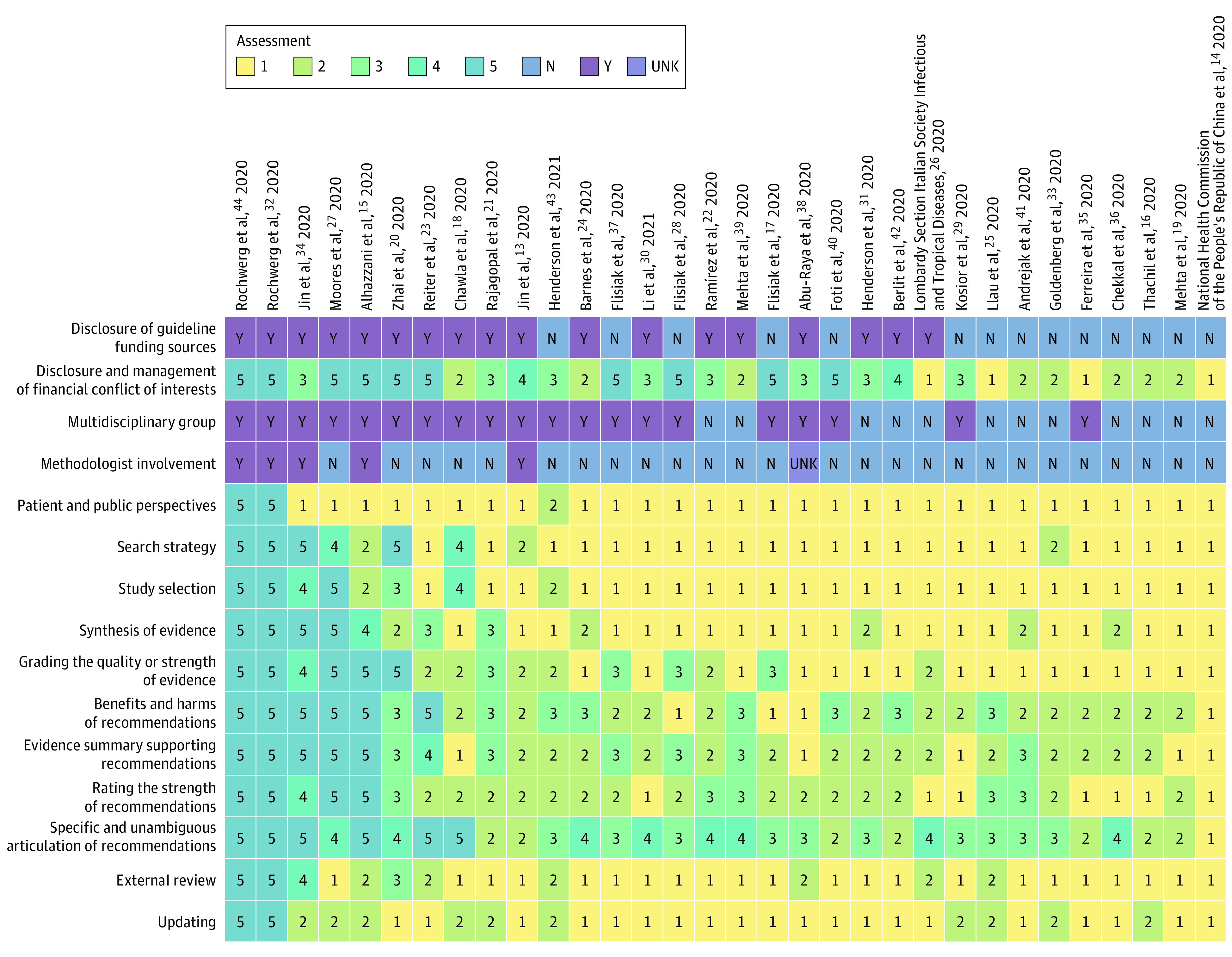
Heat Map Depicting Quality of Included Clinical Practice Guidelines The heat map depicts clinical practice guideline (CPG) quality. The CPGs are ordered from highest quality to lowest quality (left to right). We assigned a score of 5 for yes (Y) responses (3 National Guideline Clearinghouse Extent of Adherence to Trustworthy Standards [NEATS] questions) and 0 for no (N) or unknown (UKN) responses (2 NEATS questions).

We depicted quality scores for each element of the NEATS instrument for each included CPG using a Coxcomb chart (Figure 1). We illustrated the quality ratings of all included CPGs using a heat map (Figure 2). To summarize binary and categorical data in the NEATS assessment in both figures, we assigned a score of 5 for yes and 0 for no or unknown responses.

## Results

### CPG Identification and Quality Assessment

From 2226 citations, the reviewers screened 51 full texts and identified 32 eligible CPGs.^13,14,15,16,17,18,19,20,21,22,23,24,25,26,27,28,29,30,31,32,33,34,35,36,37,38,39,40,41,42,43,44^ We excluded 19 CPGs (eFigure in Supplement 1).^45,46,47,48,49,50,51,52,53,54,55,56,57,58,59,60,61,62,63^ Of the 32 included CPGs, 3 (9.4%)^15,38,40^ reported on pharmacologic treatments for critically ill patients specifically, and the remainder reported on treatments for hospitalized patients with COVID-19. Fifteen CPGs (46.9%) were international^15,16,20,21,23,24,27,31,32,33,34,36,38,43,44^ and 17 (53.1%) were national.^13,14,17,18,19,22,25,26,28,29,30,35,37,39,40,41,42^ Most CPGs (25 [78.1%]) had sponsorship from 1 or more national societies, and few CPGs (3 [9.4%])^14,23,34^ had sponsorship from government, nongovernment, or not-for-profit agencies (4 [12.5%]).^13,32,38,44^ Seven CPGs (21.9%)^13,20,23,30,31,32,34^ explicitly reported their funding sources (eTable 1 in Supplement 1). Guidelines predominantly included authors from a single WHO region (20 [62.5%]) and mostly emanated from America or Europe (Table 1). Overall, few CPGs met most of the NAM standards for trustworthiness as assessed by the NEATS instrument (Figure 1 and eTable 2 in Supplement 1).

**Table 1.** CPG Authorship by WHO Region^a^

Source	WHO region						
	Africa	Americas	Southeast Asia	Europe	Eastern Mediterranean	Western Pacific
Jin et al,^13^ 2020	No	Yes	No	No	No	Yes
National Health Commission of the People's Republic of China,^14^ 2020	No	No	No	No	No	Yes
Alhazzani et al,^15^ 2020	No	Yes	No	Yes	Yes	Yes
Thachil et al,^16^ 2020	No	Yes	No	Yes	No	Yes
Flisiak et al,^17^ 2020	No	No	No	Yes	No	No
Chawla et al,^18^ 2020	No	No	Yes	No	No	No
Mehta et al,^19^ 2020	No	No	Yes	No	No	No
Zhai et al,^20^ 2020	No	No	No	Yes	No	Yes
Rajagopal et al,^21^ 2020	No	Yes	No	Yes	No	No
Ramírez et al,^22^ 2020	No	No	No	Yes	No	No
Reiter et al,^23^ 2020	No	Yes	No	Yes	No	No
Barnes et al,^24^ 2020	No	Yes	No	No	No	No
Llau et al,^25^ 2020	No	No	No	Yes	No	No
Lombardy Section Italian Society Infectious and Tropical Diseases,^26^ 2020	No	No	No	Yes	No	No
Moores et al,^27^ 2020	No	Yes	No	Yes	No	No
Flisiak et al,^28^ 2020b	No	No	No	Yes	No	No
Kosior et al,^29^ 2020	No	No	No	Yes	No	No
Li et al,^30^ 2021	No	No	No	No	No	Yes
Henderson et al,^31^ 2020	No	Yes	No	No	No	No
Rochwerg et al,^32^ 2020	Yes	Yes	No	Yes	No	Yes
Goldenberg et al,^33^ 2020	No	Yes	Yes	Yes	Yes	Yes
Jin et al,^34^ 2020	No	Yes	No	No	No	Yes
Ferreira et al,^35^ 2020	No	Yes	No	No	No	No
Chekkal et al,^36^ 2020	Yes	No	No	No	No	No
Flisiak et al,^37^ 2020	No	No	No	Yes	No	No
Abu-Raya et al,^38^ 2020	No	Yes	No	Yes	No	Yes
Mehta et al,^39^ 2020	No	No	Yes	No	No	No
Foti et al,^40^ 2020	No	No	No	Yes	No	No
Andrejak et al,^41^ 2021	No	No	No	Yes	No	No
Berlit et al,^42^ 2020	No	No	No	Yes	No	No
Henderson et al,^43^ 2021	No	Yes	No	No	No	No
Rochwerg et al,^44^ 2020	Yes	Yes	Yes	Yes	Yes	Yes

Abbreviations: CPG, clinical practice guideline; WHO, World Health Organization.

^a^ CPGs are presented in chronologic order based on first date of publication online or in print.

### Funding and Panel Composition

Eighteen CPGs (56.3%) explicitly disclosed funding information.^13,15,18,20,21,22,23,24,26,27,30,31,32,34,38,39,42,44^ Twenty CPGs (62.5%)^13,15,17,18,20,21,23,24,27,28,29,30,32,34,35,37,38,40,43,44^ included multidisciplinary guideline panels, and 5 guideline panels (15.6%)^13,15,32,34,44^ included a methodologist.

### Disclosure of COIs and Inclusion of Patient and Public Perspectives

We identified 12 CPGs (37.5%)^13,15,17,20,23,27,28,32,37,40,42,44^ with high adherence (score of 4 or 5) to the NAM standard to disclose actual or potential financial COIs and report how COIs were incorporated or managed in the CPG development process. Eight CPGs (25.0%)^21,22,29,30,31,34,38,43^ had intermediate adherence (score of 3) and 12 CPGs (37.5%)^14,16,18,19,24,25,26,33,35,36,39,41^ had low adherence (score of 1 or 2) to this NAM standard.

Only 2 CPGs (6.3%)^32,44^ adhered (both with a score of 5) to the requirement to seek the views of patients, surrogates, advocates, and/or the public who represent those that have experience with the disease, its treatment, or its complications or those who could be affected by the CPG. These individuals could be integrated into the CPG development group or engaged in other ways or at various points in CPG development.

### Inclusion of a Systematic Review of the Evidence

Six CPGs (18.8%)^18,20,27,32,34,44^ adhered (score of 4 or 5) to the requirement to describe their search strategy in detail, including a listing of the databases searched, summary of the search terms used, and the start and end date covered by the search. Five CPGs (15.6%)^18,27,32,34,44^ adhered (score of 4 or 5) to the requirement to describe the study selection, including the number of studies identified and a summary of inclusion and exclusion criteria. Five CPGs (15.6%)^15,27,32,34,44^ adhered (score of 4 or 5) to the requirement to provide a synthesis of the evidence from the selected studies in the form of a detailed description of the body of evidence or as evidence tables or both.

### Recommendations

Six CPGs (18.8%)^15,20,27,32,34,44^ adhered (score of 4 or 5) to the requirement to provide a grade or rating of the level of confidence or certainty in the quality or strength of the evidence underpinning each recommendation. Similarly, 6 CPGs (18.8%)^15,23,27,32,34,44^ adhered (all with a score of 5) to the requirement to provide a clear description of the potential benefits and harms and link this information to specific recommendations. The same 6 CPGs^15,23,27,32,34,44^ adhered (score of 4 or 5) to the requirement to have an explicit link to a summary of the relevant evidence and link this information directly to recommendations. Five CPGs (15.6%)^15,27,32,34,44^ adhered (score of 4 or 5) to the standard to rate the strength of the recommendations (strong or conditional/weak) based on a clear and well-described evidence-to-recommendation scheme that took into account the balance between benefits and harms, available evidence, and their confidence in the underlying evidence (quantity, quality, and consistency). Fourteen CPGs (43.8%)^15,18,20,22,23,24,26,27,30,32,34,36,39,44^ adhered (score of 4 or 5) to the requirement to make specific and unambiguous recommendations that stated which actions should or should not be taken in specific situations or populations, and, where recommendations were vague or underspecified, clearly described the rationale for making recommendations.

### External Review and Plans for Updating

Only 3 CPGs (9.4%)^32,34,44^ adhered (score of 4 or 5) to the requirement to describe an external review process by specifying (name and description) relevant stakeholders (ie, scientific and clinical experts, organizations, agencies, patients, and representatives) and a process for external review. Only 2 CPGs (6.3%; both with a score of 5)^32,44^ had a prespecified procedure to update the CPG that included the time frame for updating, the process by which a decision would be made to update the CPG, and a description of how the update would be conducted.

We depict the total NEATS score for each included CPG in a heat map in Figure 2. Common features of the highest-quality CPGs (n = 5)^15,27,32,34,44^ were that they were multidisciplinary and included collaborators from at least 2 WHO regions. Four of these 5 highest-quality CPGs^15,32,34,44^ included a methodologist in their guideline panel.

### Direction of Recommendations for Pharmaceutical Interventions

Table 2 depicts the evolution and direction of recommendations over time for each pharmacologic intervention reported by 3 or more CPGs in chronologic order. Clinical practice guidelines consistently recommended or suggested use of supportive (ie, vasopressors, inotropes) and prophylactic treatments (venous thromboembolism or deep venous thrombosis prophylaxis, histamine receptor antagonists, or proton pump inhibitors) for hospitalized patients with COVID-19. Notwithstanding, we noted relatively inconsistent recommendations for most pharmacologic treatments identified (empirical antibiotics, azithromycin, corticosteroids, hydroxychloroquine or chloroquine, lopinavir or ritonavir, remdesivir, tocilizumab, interferon, favipiravir, and oseltamivir) in the included CPGs.

**Table 2.** Direction of Recommendations for Pharmacologic Treatments of Hospitalized Patients With COVID-19^a^

Source	VTE/DVT prophylaxis	Empirical antibiotics	Azithromycin	Histamine receptor antagonist/PPI	Vasopressors/inotropes	Oral or IV corticosteroids	Hydroxy- chloroquine/choloroquine	Lopinavir/ritonavir	Remdesivir	Tocilizumab	Interferon	Favipiravir	Oseltamivir
Jin et al,^13^ 2020	R	NR	NR	R	R	R	NR	R	NR	NR	R	NR	NR
National Health Commission of the People's Republic of China,^14^ 2020	NR	NR	NR	NR	NR	R	R	R	NR	R	R	NR	NR
Alhazzani et al,^15^ 2020	NR	R	NR	NR	R	R/NR/X^b^	NR	X	NR	NR	NR	NR	NR
Thachil et al,^16^ 2020	R	NR	NR	NR	NR	NR	NR	NR	NR	NR	NR	NR	NR
Flisiak et al,^17^ 2020	NR	NR	NR	NR	NR	R	R	R	R	R	NR	R	R
Chawla et al,^18^ 2020	NR	NR	NR	NR	NR	X	X	NR	NR	NR	NR	NR	NR
Mehta et al,^19^ 2020	R	R	NR	R	R	X	R	R	R	NR	NR	NR	R
Zhai et al,^20^ 2020	R	NR	NR	NR	NR	NR	NR	NR	NR	NR	NR	NR	NR
Rajagopal et al,^21^ 2020	R	NR	NR	NR	NR	NR	R	NR	R	NR	NR	NR	NR
Ramírez et al,^22^2020	R	NR	NR	NR	NR	NR	NR	NR	NR	NR	NR	NR	NR
Reiter et al,^23^ 2020	NR	NR	NR	NR	NR	NR	R	NR	NR	NR	NR	NR	NR
Barnes et al,^24^ 2020	R	NR	NR	NR	NR	NR	NR	NR	NR	NR	NR	NR	NR
Llau et al,^25^ 2020	R	NR	NR	NR	NR	NR	NR	NR	NR	NR	NR	NR	NR
Lombardy Section Italian Society Infectious and Tropical Diseases,^26^ 2020	NR	NR	NR	NR	NR	R	R	R	R	R	NR	NR	R
Moores et al,^27^ 2020	R	NR	R	NR	NR	NR	NR	NR	NR	NR	NR	NR	NR
Flisiak et al,^28^ 2020	R	R	X	NR	NR	R	R	R	R	R	NR	X	X
Kosior et al,^29^ 2020	R	NR	NR	NR	NR	NR	NR	NR	NR	NR	NR	NR	NR
Li et al,^30^ 2021	R	NR	NR	NR	NR	NR	NR	NR	NR	NR	NR	NR	NR
Henderson et al,^31^ 2020	R	NR	NR	NR	NR	R	NR	NR	NR	R	NR	NR	NR
Rochwerg et al,^32^ 2020	NR	NR	NR	NR	NR	NR	NR	NR	R	NR	NR	NR	NR
Goldenberg et al,^33^ 2020	R	NR	NR	NR	NR	NR	NR	NR	NR	NR	NR	NR	NR
Jin et al,^34^ 2020	R	NR	NR	NR	R	R/NR/X^c^	NR	X	R	NR	R	R	NR
Ferreira et al,^35^ 2020	NR	NR	NR	NR	NR	R	NR	NR	NR	NR	NR	NR	NR
Chekkal et al,^36^ 2020	R	NR	NR	NR	NR	NR	NR	NR	NR	NR	NR	NR	NR
Flisiak et al,^37^ 2020	R	X	X	NR	NR	R	X	X	R	R	NR	X	X
Abu-Raya et al,^38^ 2020	R	NR	NR	NR	NR	R	NR	NR	NR	NR	NR	NR	NR
Mehta et al,^39^ 2020	R	NR	X	NR	NR	R	X	X	R	X	X	NR	NR
Foti et al,^40^ 2020	R	X	NR	NR	R	R	NR	NR	NR	NR	NR	NR	NR
Andrejak et al,^41^ 2021	NR	NR	NR	NR	NR	R	NR	NR	NR	NR	NR	NR	NR
Berlit et al,^42^ 2020	NR	NR	NR	NR	NR	R	NR	NR	NR	NR	NR	NR	NR
Henderson et al,^43^ 2021	R	NR	NR	NR	NR	R	NR	NR	NR	X	NR	NR	NR
Rochwerg et al,^44^ 2020	NR	NR	NR	NR	NR	R**/**NR/X^d^	X	X	X	NR	NR	NR	NR

Abbreviations: DVT, deep venous thrombosis; IV, intravenous; NR, no recommendation; PPI, proton pump inhibitor; R, recommend/suggest use for patients hospitalized or in the intensive care unit with COVID-19; VTE, venous thromboembolism; X, recommend/suggest against use for patients hospitalized or in the intensive care unit with COVID-19.

^a^ Clinical practice guidelines are presented in chronologic order based on first date of publication online or in print.

^b^ For adults with COVID-19 and refractory shock, we suggest using low-dose corticosteroid therapy (shock-reversal) compared with no corticosteroids. In adults with COVID-19 and adult respiratory distress syndrome undergoing mechanical ventilation, we suggest using systemic corticosteroids compared with no corticosteroids. In adults with COVID-19 and respiratory failure (without adult respiratory distress syndrome) undergoing mechanical ventilation, we suggest against the routine use of systemic corticosteroids.

^c^ When the condition of patients with severe or critical COVID-19 deteriorates dramatically, low-dose glucocorticoids with a short course may be considered (grade 2B). We do not suggest glucocorticoids for patients with COVID-19 in general (grade 2B).

^d^ A strong recommendation for systemic corticosteroids in patients with severe and critical COVID-19. A conditional recommendation against systemic corticosteroids in patients with nonsevere COVID-19.

Clinical practice guideline recommendations evolved during the period of our review to recommend or suggest the use of corticosteroids for hospitalized patients with COVID-19 (Table 2). Conversely, CPGs evolved from largely recommending or suggesting use of hydroxychloroquine or chloroquine, lopinavir or ritonavir, remdesivir, and tocilizumab to recommending or suggesting against their use for hospitalized patients with COVID-19 during the period covered by our review.

## Discussion

In this systematic review of CPGs evaluating pharmacologic treatments for hospitalized patients with COVID-19, we found that few CPGs met the NAM standards for trustworthiness as assessed by the NEATS instrument.^1,11^ Although nearly two-thirds of CPGs included multidisciplinary guideline panels, fewer than 20% of CPG panels included a methodologist. Only 37.5% of CPGs had a detailed disclosure of actual or potential COIs. Few CPGs (6.3%) included patient and public perspectives. Fewer than 20% of included COVID-19–related CPGs described their search strategy, a process for study selection, or provided a synthesis of the evidence. Although nearly half of CPGs made suggestions or recommendations for or against treatments, fewer than 20% of CPGs provided a grade or rating of the level of confidence in or certainty with the quality or strength of the evidence, offered a clear description of the potential benefits and harms with links to specific recommendations, or rated the strength of the recommendations using a clear grading scheme. Fewer than 10% of CPGs underwent external review and even fewer described a process for updating. The overall quality of CPGs, as assessed by the NEATS score, was low. Multidisciplinary panels that included a methodologist and collaborators from at least 2 WHO regions were features of high-quality COVID-19 CPGs.

The rate at which CPGs pertaining to the management of COVID-19 in various settings (outpatient, inpatient, or intensive care unit) have been published is unprecedented. During a pandemic specifically, there is a high demand for early, systematically developed statements that reflect best practices based on available evidence to guide the practice of health care professionals. Nonetheless, strong methodologic standards for CPGs are essential to avoid promulgating useless or potentially harmful treatments and wasting health care resources.^64^ Overall, most included CPGs in our study failed to meet NAM standards and consequently were at increased risk of bias. Although producing high-quality guidelines may be viewed as impractical during a pandemic, this review identified features of high-quality COVID-19–related CPGs using the NEATS instrument. Although most high-quality CPGs tended to be published later in the pandemic, a high-quality CPG in our review was published in March 2020.^15^ Moreover, we noted that updates of CPGs published earlier in the pandemic tended to be of higher quality than the parent documents (Figure 2). Improvement of CPG quality over time may reflect accumulating knowledge, clinical experience, or lead-time bias.

Our findings align with other assessments of nonpandemic and pandemic CPG quality.^12,64,65,66^ From 130 randomly selected CPGs from the National Guideline Clearinghouse, Kung et al^65^ found that the median number of NAM standards satisfied was 8 of 18 (44.4% [IQR, 36.1%-52.8%]). The authors noted that fewer than half of their included CPGs and one-third of CPGs produced by subspecialty societies met more than 50% of the NAM standards.^65^ Similar to our study, others have shown that fewer than half of CPGs provided information regarding COIs,^12,65^ few CPGs included patients or patient representatives,^12,64,65,66^ and the CPGs rarely included a process for updating.^64,66^ The present review adds to the literature by documenting that fewer than 20% of CPGs included a systematic review or adhered to the International Organization for Standardization to generate recommendations for care. Although several CPGs in our review (14 of 32 [43.8%]) made suggestions or recommendations for or against treatments, they infrequently provided a grade or rating of the level of confidence or certainty regarding the quality or strength of the evidence (6 of 32 [18.8%]), offered a clear description of the potential benefits and harms (6 of 32 [18.8%]), or rated the strength of the recommendations using a clear grading scheme (5 of 32 [15.6%]). As such, the guidance statements from most CPGs included in our review were not optimally informed by the key dimensions of evidence on pharmacologic interventions for COVID-19. Contrary to a review of oncology CPGs,^66^ most CPGs in our review did not undergo external peer review. Similar to our study, an earlier review of 19 COVID-19–specific CPGs^12^ found that the overall quality of CPGs was poor; lacked detail; had inconsistent recommendations, even for the same intervention; and did not provide explicit linkage between the evidence and generating recommendations. Recently, Stamm et al^64^ evaluated the quality of 188 general COVID-19 CPGs published from February 1 to April 27, 2020, using the AGREE II tool. The CPGs in this review were largely (83%) based on informal consensus without clear evidence summaries and scored highest for scope and purpose (89%) and lowest (25%) for rigor of development. The latter finding may relate to the paucity of evidence available early in the pandemic. Unlike previous COVID-19 CPG reviews,^12,64^ we limited our review to pharmacologic treatments for COVID-19, included CPGs with broad potential reach (authored by societies and government or nongovernment organizations), and appraised quality using the NEATS (vs AGREE II) instrument. Taken together, systematic reviews of CPG quality have identified that most CPGs were of low overall methodologic quality and tended to make recommendations that promoted more interventions as opposed to more effective interventions*.*

Instruments that appraise CPG quality provide stakeholders with a metric to evaluate and select the most rigorously developed CPGs with the goal of improving patient care, safety, and outcomes. The AGREE II checklist focuses on assessment of the quality and reporting of CPGs in 6 domains (scope and purpose, stakeholder involvement, rigor of development, clarity of presentation, applicability, and editorial independence) but does not address the clinical validity of CPG recommendations. By contrast, the 15-item NEATS instrument assesses adherence to NAM standards. The NEATS tool has been shown to have high interrater reliability (weighted κ = 0.73) and external validity.^11^ To ensure consistency and reliability of judgments, 2 trained personnel assess each CPG using the NEATS tool at the National Guideline Clearinghouse. Subsequently, these assessments are shared with CPG developers to enhance the accuracy and completeness of NEATS quality summaries. This feedback loop provides guideline developers with a benchmark to compare their processes against the NAM standards and an opportunity to clarify their methods.^67^ Several authors^64,68^ have noted that the additional rigor required to adhere to these standards may come at the cost of increased complexity, expertise, money, and time to CPG completion, most of which are in short supply during a pandemic. Future research is needed to compare appraisal tools, understand how to create CPGs that are ready for implementation, and aid stakeholders (clinicians, patients, and the public) to be informed CPG consumers.

Several strategies might enhance the development of trustworthy CPGs, even in the setting of a pandemic. First, CPG panels should include participation of a methodologic expert (eg, an epidemiologist, biostatistician, health services researcher). Their expertise adds to decisions regarding study design and the potential for bias and influence on study findings, methods to minimize bias in the conduct of systematic reviews, use of quantitative methods, conduct of qualitative synthesis, and issues related to data collection and management.^11^ Second, approaches that prioritize broad collaborations that engage multidisciplinary stakeholders who work together and share expertise and resources and are from at least 2 WHO regions may be optimal and lead to the production of fewer but higher-quality CPGs that are poised for updates as new evidence emerges. This approach could not only limit duplication of efforts but also limit publication of inconsistent recommendations. As opposed to de novo CPG development, local and regional groups should consider appraising and adapting existing high-quality CPGs to their practice context using the ADAPTE process.^69^ This 3-stage process includes start-up (assessment of skills and resources required), adaptation (selection of specific questions and CPG retrieval, quality assessment, selection, and compilation), and end stage (seeking opinions of decision makers affected by CPG, CPG revision, and finalization).^69^ Inherent to the ADAPTE process is access to CPGs and availability of local expertise in CPG appraisal. Adaptation to the clinical context is an important consideration, because most CPGs in our review were sponsored by societies with infrastructure and expertise and few were developed in low- and middle-income countries. Third, journal editors and peer reviewers should mandate use of 1 or more CPG appraisal tools at the time of manuscript submission to ensure publication of high-quality and trustworthy CPGs.

### Strengths and Limitations

Our review has several strengths. Unlike prior reviews of COVID-19–related CPGs, we limited our review to pharmacologic treatments for hospitalized patients with COVID-19, included CPGs with broad potential reach (sponsored by societies and government or nongovernment organizations), and appraised CPG quality using the NEATS (vs AGREE II) instrument. To our knowledge, this is the first report to use the NEATS instrument to appraise CPG quality outside of the National Guideline Clearinghouse. We prioritized use of the NEATS tool because it had undergone rigorous development and testing and is aligned with NAM standards for trustworthy CPGs. We performed a comprehensive search and reviewed citations, abstracted data, and assessed quality in duplicate to limit ascertainment bias.

Our review also has some limitations. First, we limited our search to CPGs that were published in peer-reviewed journals. Second, we did not contact CPG authors to verify the methodologic aspects of their respective guidelines. Consequently, our assessment of methodologic expertise may be an underestimate, limited by reporting of this information in CPGs. Third, we did not have specific information pertaining to whether the included CPGs underwent peer review (regular, expedited, or absent) or were appraised using a quality checklist or other tool by authors at the time of submission. Notwithstanding, these points highlight the need for high publication standards even in the unique circumstances posed by a pandemic.

## Conclusions

Few COVID-19 CPGs met NAM standards for trustworthy guidelines. Approaches that prioritize engagement of a methodologist and multidisciplinary collaborators from at least 2 WHO regions may lead to the production of fewer, high-quality CPGs that are poised for updates as new evidence emerges.

## Notes

Supplement 1. **eMethods.** NEATS Instrument **eFigure.** Identification of Clinical Practice Guidelines (CPGs) **eTable 1.** Characteristics of Included Clinical Practice Guidelines (CPGs) **eTable 2.** Summary of NEATS Scores for the Included Clinical Practice Guidelines (CPGs)

Supplement 2. **Nonauthor Collaborators.** The Academy of Critical Care: Development, Evaluation, and Methodology (ACCADEMY)
