# Supplementary material for: Adherence of Clinical Practice Guidelines for Pharmacologic Treatments of Hospitalized Patients With COVID-19 to Trustworthy Standards: A Systematic Review
Source: JAMA Netw Open. 2021 Dec 10;4(12):e2136263. doi: 10.1001/jamanetworkopen.2021.36263 (PMC8665373; doi:10.1001/jamanetworkopen.2021.36263)
Supplement: Supplement 1. — eMethods. NEATS Instrument eFigure. Identification of Clinical Practice Guidelines (CPGs) eTable 1. Characteristics of Included Clinical Practice Guidelines (CPGs) eTable 2. Summary of NEATS Scores for the Included Clinical Practice Guidelines (CPGs) [file jamanetwopen-e2136263-s001.pdf]

Supplementary Online Content

Burns KEA, Laird M, Stevenson J, et al; Academy of Critical Care: Development, Evaluation, and Methodology (ACCADEMY). Adherence of clinical practice guidelines for pharmacologic treatments of hospitalized patients with COVID-19 to trustworthy standards: a systematic review. *JAMA Netw Open*. 2021;4(12):e2136263. doi:10.1001/jamanetworkopen.2021.36263

eMethods. NEATS Instrument
eFigure. Identification of Clinical Practice Guidelines (CPGs)
eTable 1. Characteristics of Included Clinical Practice Guidelines (CPGs)
eTable 2. Summary of NEATS Scores for the Included Clinical Practice Guidelines (CPGs)

This supplementary material has been provided by the authors to give readers additional information about their work.

© 2021 Burns KEA et al. *JAMA Network Open*.

eMethods. NEATS Instrument

Part A. National Guideline Clearinghouse and National Quality Measures Clearinghouse Editorial Board and Expert Panel Members

Editorial Board from July 2013 to July 2017

Richard C. Hermann, MD, MS (Co-chair)
Paul G. Shekelle, MD, PhD, MPH (Co-chair)
Rhonda Anderson, Rn, DNSC(h), FAAN, FACHE
Cheryl Damberg, PhD, MPH
Peggy Honore, DHA
Lisa Lang, MPP
Kathleen N. Lohr, PhD
Bernadette Mazurek Melnyk, PhD, RN, CPNP/PMHNP, FAANP, FNAP, FAAN
Susan L. Norris, MD, MSc, MPH
Craig Robbins, MD, MPH
Eric C. Schneider, MD, MSc
Richard N. Shiffman, MD, MCIS

Expert Panel 2011 to July 2013

Rhonda Anderson, RN, DNSc(h), FAAN, FACHE
Lisa Bero, PhD
George Bo-Linn, MD, MHA, FACP
John Clarke, MD
Kathyrn Coltin, MPH
Dave Davis, MD, CCFP, FCFP
Duane Davis, MD
A. Mark Fendrick, MD
Raymond J. Gibbons, MD
Richard Van Harrison, PhD
Gordon Hunt, MD
George Isham, MD, MS
Richard Justman, MD
Vincent Kerr, MD
Timothy F. Kresowik, MD, MS
Robert Krughoff, JD
Lisa Lang, MPP
Susan Norris, MD, MPH, MSc
Douglas Owens, MD, MS
L. Gregory Pawlson, MD, MPH
Michael Rapp, MD, JD, FACEP
Richard Shiffman, MD, MCIS
Jeffrey Thompson, MD
Marita Titler, PhD, RN, FAAN
Diana Torres-Burgos, MD, MPH
Paul Wallace, MD
Jed Weissberg, MD
Josie Williams, MD, MMM, CPE
Modena Wilson, MD, MPH

© 2021 Burns KEA et al. *JAMA Network Open*.

Part B. National Guideline Clearinghouse Extent Adherence to Trustworthy Standards (NEATS) Instrument

The numbered domain items that follow reflect standards from the Institute of Medicine (IOM) report *Clinical Practice Guidelines We Can Trust.* The standard from the IOM report is listed in the first box of each domain item, and it is the principle that underpins the actual rating criteria that appear in the box immediately underneath, which is highlighted light-green. For several domain items, the rating criteria are based on the IOM principle but take either a broader or a more simplified approach. Although we value the IOM standards for their ambition, comprehensiveness, and attention to detail, we tailored the rating criteria as necessary for practical implementation of the NEATS Instrument for assessing the many guidelines represented on the NGC Web site.

The stated rating criteria are what raters should consider when selecting the response; response options are either Yes/No or points on a Likert scale of 1 to 5. For the scale, 1 reflects the least adherence to the criteria listed and 5 reflects the most adherence to the criteria listed.

1. Disclosure of Guideline Funding Source

**Reference IOM Standard**<br>*The processes by which a clinical practice guideline (CPG) is funded should be detailed explicitly and publicly accessible.*
**Please rate on this criterion:**<br><br>**The clinical practice guideline (CPG) discloses and states explicitly its funding source.**

Please review the description and guidance below and then choose one option:

**YES**	**NO**
◯	◯

Description

This standard asks for information regarding the funding of the guideline's development. Implicit in this standard is the notion that transparency of funding "gives users confidence that guidelines are… largely free from bias… and therefore trustworthy." (IOM 2011, p. 77) The clinical practice guideline (CPG) or supporting documents should list the funding source(s) for its development. This information should be publically available.

© 2021 Burns KEA et al. *JAMA Network Open*.

2. Disclosure and Management of Financial Conflicts of Interests (COIs)

Reference IOM Standard

- *Prior to selection of the guideline development group (GDG), individuals being considered for membership should declare all interests and activities potentially resulting in COI with development group activity, by written disclosure to those convening the GDG. Disclosure should reflect all current and planned commercial (including services from which a clinician derives a substantial proportion of income), non-commercial, intellectual, institutional, and patient–public activities pertinent to the potential scope of the CPG.*
- *Disclosure of COIs within GDG: All COI of each GDG member should be reported and discussed by the prospective development group prior to the onset of his or her work. Each panel member should explain how his or her COI could influence the CPG development process or specific recommendations.*

Please rate on this criterion:

Financial conflicts of interest of guideline development group (GDG) members have been disclosed and managed.

Please review the description and guidance below and then choose one option:

Lowest Adherence				Highest Adherence
1	2	3	4	5
○	○	○	○	○

Description

This standard addresses the issue of actual or potential relationships between members of the GDG and entities, commercial or otherwise, with financial or intellectual interests in the CPG topic.

The CPG or supporting documents should provide a detailed disclosure of actual or potential financial COIs of each GDG member AND if any COIs are present, the document should describe how these conflicts may have affected the guideline process and any steps taken to manage and minimize their effect (e.g., recusal, divestment).

© 2021 Burns KEA et al. *JAMA Network Open*.

3a. Guideline Development Group (GDG) Composition: Multidisciplinary

**Reference IOM Standard**<br>*The GDG should be multidisciplinary and balanced, comprising a variety of methodological experts, clinicians, and populations expected to be affected by the CPG.*
**Please rate on this criterion:**<br><br>**The guideline development group (GDG) includes individuals from a variety of relevant clinical specialties and other professional groups.**

Please review the description and guidance below and then choose one option:

**YES**	**NO**	**UNKNOWN**
◯	◯	◯

Description

This standard seeks to reduce the potential for bias that can sometimes result from a homogeneous GDG by encouraging a GDG comprising members from multiple disciplines. While each CPG will have a different set of clinical specialties that are relevant, a multidisciplinary GDG can include subject matter experts from a variety of professional backgrounds, paraprofessionals, statisticians, program managers, and members of the public. The GDG is multidisciplinary if more than one relevant clinical specialty is represented, based on stated disciplines (e.g., it includes representatives of more than one clinical specialty or professional group). This includes the situation when a GDG member is a nonclinical specialist.

© 2021 Burns KEA et al. *JAMA Network Open*.

3b. Guideline Development Group (GDG) Composition: Methodologist

**Reference IOM Standard**<br>*The GDG should be multidisciplinary and balanced, comprising a variety of methodological experts, clinicians, and populations expected to be affected by the CPG.*
**Please rate on this criterion:**<br><br>**The guideline states that it included a methodological expert in the guideline development group (GDG) and it identifies the methodologist.**

Please review the description and guidance below and then choose one option:

**YES**	**NO**	**UNKNOWN**
◯	◯	◯

Description

This standard seeks to ensure that the guideline was developed with the participation of a methodological expert. As described by the IOM, "methodologists (e.g., epidemiologists, biostatisticians, health services researchers) perform much of the research on the conduct of systematic reviews (SRs) and are likely to stay up-to-date with the literature on methods. Their expertise includes decisions about study design and potential for bias and influence on findings, methods to minimize bias in the SR, qualitative synthesis, quantitative methods, and issues related to data collection and data management." (IOM [Institute of Medicine]. *Finding What Works in Health Care: Standards for Systematic Reviews.* Washington (DC): The National Academies Press. 2011)

The CPG or supporting documents should make clear that methodologists were involved in the CPG development process, specifically listing methodologists and detailing their specific roles.

© 2021 Burns KEA et al. *JAMA Network Open*.

4. Patient and Public Perspectives

**Reference IOM Standard**<br>*Patient and public involvement should be facilitated by including (at least at the time of clinical question formulation and draft CPG review) a current or former patient, and a patient advocate or patient/consumer organization representative in the GDG.*
**Please rate on this criterion:**<br><br>**The guideline development group (GDG) sought the views, perspectives, and preferences of patients, patient surrogates (parents, caretakers), patient advocates, and/or the public intended to represent those who have experience with the disease, its treatments, or complications, or those who could be affected by the guideline.**

Please review the description and guidance below and then choose one option:

**Lowest Adherence**				**Highest Adherence**
**1**	**2**	**3**	**4**	**5**
◯	◯	◯	◯	◯

Description

This standard seeks that the perspectives of the target population be included in the guideline development process. The target population includes patients, patient surrogates (parents, caregivers), patient advocates, and/or the public, i.e., those who have experience with the disease, its treatments, or complications, or those who could be impacted by the guideline. While the original IOM standard prioritizes patient or surrogate representation in the GDG, we have broadened our assessment to encompass incorporation of patient perspectives in other ways and at various points in the guideline development process, as well.

Inclusion of patient perspective can take many forms: a patient representative on the GDG, consultation with patients to set priorities for topics, and external review by stakeholders, the public, or consumers, including drafts available for public comment (Please note for drafts for public comment, it must be clear that public comment specifically involved patients and that those comments were addressed). In addition, incorporating literature published on patient preferences and perspectives that relate to the guideline's recommended care is also acceptable.

The GDG or companion documents should include at least one patient, surrogate (parents, caretakers) or advocate AND the CPG should be clear about how those individuals contributed (e.g., clinical question formulation, review of draft CPG). If utilized, the CPG should also provide detailed information about how patient perspectives (i.e., studies regarding patient preference) were incorporated.

© 2021 Burns KEA et al. *JAMA Network Open*.

5a. Use of a Systematic Review of Evidence – the Search Strategy

**Reference IOM Standard**
Clinical practice guideline developers should use systematic reviews that meet standards set by the Institute of Medicine's Committee on Standards for Systematic Reviews of Comparative Effectiveness Research.
**Please rate on this criterion:**<br><br>**The CPG or a related companion document describes a search strategy that includes a listing of database(s) searched, a summary of search terms used, the specific time period covered by the literature search including the beginning date (month/year) and end date (month/year).**

Please review the description and guidance below and then choose one option:

**Lowest Adherence**				**Highest Adherence**
**1**	**2**	**3**	**4**	**5**
○	○	○	○	○

Description
This standard expects that guidelines based on a systematic review of the evidence describe in detail the search strategy. This should include a listing of database(s) searched, a summary of search terms used, the specific time period covered by the literature search including the beginning date (month/year) and end date (month/year).

The CPG or companion documents should provide a detailed description of the search strategy that includes a listing of database(s) searched, a summary of search terms used, the specific time period covered by the literature search including the beginning date (month/year) and end date (month/year). The information should be well-described and complete, with multiple databases searched and specific search terms. The CPG may include additional details such as extensive search terms, MeSH terms, key questions, or other specific details of the search strategy.

© 2021 Burns KEA et al. *JAMA Network Open*.

5b. Use of a Systematic Review of Evidence – the Study Selection

**Reference IOM Standard**<br>*Clinical practice guideline developers should use systematic reviews that meet standards set by the Institute of Medicine's Committee on Standards for Systematic Reviews of Comparative Effectiveness Research.*
**Please rate on this criterion:**<br><br>**The CPG or a related companion document describes the study selection that includes the number of studies identified, the number of studies included, and a summary of inclusion and exclusion criteria.**

Please review the description and guidance below and then choose one option:

**Lowest Adherence**				**Highest Adherence**
**1**	**2**	**3**	**4**	**5**
◯	◯	◯	◯	◯

Description

This standard expects that guidelines based on a systematic review of the evidence describe in detail the study selection. This should include the number of studies identified by search, the number of studies included, and a summary of the inclusion and exclusion criteria.

The CPG or companion documents should provide a description of study selection that includes the number of studies identified, the number of studies included, and a detailed summary of inclusion and exclusion criteria. The number of documents identified and included may be listed in the results section and may also be displayed in a flowchart (e.g., PRISMA flowchart).

© 2021 Burns KEA et al. *JAMA Network Open*.

5c. Use of a Systematic Review of Evidence – the Synthesis of Evidence

**Reference IOM Standard**<br>*Clinical practice guideline developers should use systematic reviews that meet standards set by the Institute of Medicine's Committee on Standards for Systematic Reviews of Comparative Effectiveness Research.*
**Please rate on this criterion:**<br><br>**The CPG or a related companion document provides a synthesis of evidence from the selected studies, i.e., an analysis of individual studies and the body of evidence, in the form of a detailed description or evidence tables, or both.**

Please review the description and guidance below and then choose one option:

**Lowest Adherence**				**Highest Adherence**
**1**	**2**	**3**	**4**	**5**
◯	◯	◯	◯	◯

Description

This standard expects that guidelines based on a systematic review of the evidence describe in detail the synthesis of evidence from the studies that were selected. This means that the CPG or a related companion document should include an analysis of individual studies and also an analysis the body of evidence taken as a whole. This could take the form of a detailed narrative description of the nature and quality of studies or evidence tables that capture such details about the studies, or both.

The CPG or companion documents should provide a synthesis of the evidence from the selected studies that includes well-crafted, detailed evidence tables and a thorough narrative description and discussion of the evidence.

© 2021 Burns KEA et al. *JAMA Network Open*.

6. Grading or Rating the Quality or Strength of Evidence

**Reference IOM Standard**<br>*For each recommendation, the following should be provided:*<br>• *A rating of the level of confidence in (certainty regarding) the evidence underpinning the recommendation.*
**Please rate on this criterion:**<br><br>**The CPG provides a grading or rating of the level of confidence in or certainty regarding the quality or strength of the evidence for each recommendation.**

Please review the description and guidance below and then choose one option:

**Lowest Adherence**				**Highest Adherence**
**1**	**2**	**3**	**4**	**5**
◯	◯	◯	◯	◯

Description

This standard asks that evidence be graded or rated according to a scheme that takes into account the quality of and level of confidence or certainty regarding the evidence. Note that this domain item is a grading or rating of the strength of evidence underpinning recommendations.

The CPG's recommendations should be accompanied by a grade or rating of the evidence derived from a clear and well-described scheme of the level of confidence in (or certainty regarding) the evidence. The grade or rating should be linked clearly and directly to the recommendation(s).

© 2021 Burns KEA et al. *JAMA Network Open*.

7. Benefits and Harms of Recommendations

**Reference IOM Standard**
For each recommendation, the following should be provided:<br>• *An explanation of the reasoning underlying the recommendation, including a clear description of potential benefits and harms*
**Please rate on this criterion:**<br><br>**The potential benefits and harms of recommended care are clearly described for the recommendations.**

Please review the description and guidance below and then choose one option:

**Lowest Adherence**				**Highest Adherence**
**1**	**2**	**3**	**4**	**5**
◯	◯	◯	◯	◯

Description
This standard expects developers to consider and describe explicitly the potential benefits and harms as they arrive at the CPG's recommendations. Potential harms may include risk of side effects or complications.

The CPG should describe clearly and in detail the potential benefits and harms of recommendations AND also link explicitly this information to specific recommendations.

© 2021 Burns KEA et al. *JAMA Network Open*.

8. Evidence Summary Supporting Recommendations

**Reference IOM Standard**<br>*For each recommendation, the following should be provided:*<br>• *An explanation of the reasoning underlying the recommendation, including a summary of relevant available evidence (and evidentiary gaps), description of the quality (including applicability), quantity (including completeness), and consistency of the aggregate available evidence.*
**Please rate on this criterion:**<br><br>**A summary of the relevant supporting evidence is explicitly linked to recommendations.**

Please review the description and guidance below and then choose one option:

**Lowest Adherence**				**Highest Adherence**
**1**	**2**	**3**	**4**	**5**
○	○	○	○	○

Description

This standard seeks that recommendations have an explicit link to a summary of the relevant evidence underlying the recommendation. This differs from the synthesis of the evidence in that it ties specific evidence to specific recommendations and will generally be much briefer.

The CPG or supporting documents should provide a thoughtful summary of the relevant supporting evidence (e.g., in an explicit discussion of the evidence) AND link this information directly to recommendations.

© 2021 Burns KEA et al. *JAMA Network Open*.

9. Rating the Strength of Recommendations

**Reference IOM Standard**<br>*For each recommendation, the following should be provided:*<br>• *A rating of the strength of the recommendation in light of [benefits and harms, available evidence, and the confidence in the underlying evidence].*
**Please rate on this criterion:**<br><br>**The CPG gives a rating of the strength of the recommendation for each recommendation that takes into account benefits and harms, available evidence, and the confidence in the underlying evidence.**

Please review the description and guidance below and then choose one option:

**Lowest Adherence**				**Highest Adherence**
**1**	**2**	**3**	**4**	**5**
◯	◯	◯	◯	◯

Description

This standard expects that the CPG provides a rating for key recommendations according to a scheme that takes into account the confidence in that evidence (e.g., quantity, quality, and consistency of the available evidence), and the balance of benefits and harms.

The CPG should provide a rating for the strength of each recommendation that is based on a clear and well-described grading scheme that takes into account the confidence in the evidence and the balance of benefits and harms.

© 2021 Burns KEA et al. *JAMA Network Open*.

10. Specific and Unambiguous Articulation of Recommendations

**Reference IOM Standard**<br>*Recommendations should be articulated in a standardized form detailing precisely what the recommended action is and under what circumstances it should be performed.*
**Please rate on this criterion:**<br><br>**The recommendations are specific and unambiguous, stating what action should or should not be taken in what situations and for what population groups. Where the CPG recommendations are intentionally vague or underspecified, the CPG clearly describes the rationale behind those recommendations.**

Please review the description and guidance below and then choose one option:

**Lowest Adherence**				**Highest Adherence**
**1**	**2**	**3**	**4**	**5**
◯	◯	◯	◯	◯

Description
This standard expects that a CPG's recommendations are clear, concrete, and precise to facilitate its implementation. The recommendations should not be vague or open to interpretation, but instead they should say directly what action should or should not be taken in what situations and for what population groups.

The CPG's recommendations should provide a concrete and precise description of (1) what is being recommended, (2) for whom, and (3) under which circumstances. The CPG should give a clear rationale for any intentional vagueness or under-specification.

© 2021 Burns KEA et al. *JAMA Network Open*.

11. External Review

**Reference IOM Standard**<br>*External reviewers should comprise a full spectrum of relevant stakeholders, including scientific and clinical experts, organizations (e.g., health care, specialty societies), agencies (e.g., federal government), patients, and representatives of the public.*
**Please rate on this criterion:**<br><br>**The guideline has been reviewed by relevant stakeholders, including scientific and clinical experts, organizations, agencies, and patients.**

Please review the description and guidance below and then choose one option:

**Lowest Adherence**				**Highest Adherence**
**1**	**2**	**3**	**4**	**5**
◯	◯	◯	◯	◯

Description

This standard expects that reviewers who were not involved in the guideline's development review it before publication so that the GDG can ensure "the balance, comprehensiveness, and quality" of the guideline. Reviewers can include experts in the clinical area, methodologists, and members of the public. The CPG or supporting documents should describe an external review process by specific relevant stakeholders who are outside the guideline development process and organization. This can include scientific and clinical experts, health care specialty societies, public sector agencies, and patients. Stakeholders should be named or types of stakeholders described, and the process of external review should be described.

© 2021 Burns KEA et al. *JAMA Network Open*.

12. Updating

**Reference IOM Standard**<br>*The CPG publication date, date of pertinent systematic evidence review, and proposed date for future CPG review should be documented in the CPG.*
**Please rate on this criterion:**<br><br>**The CPG describes a procedure to update the guideline.**

Please review the description and guidance below and then choose one option:

**Lowest Adherence**				**Highest Adherence**
**1**	**2**	**3**	**4**	**5**
○	○	○	○	○

Description
This standard expects that the developers have a process in place to keep the guideline current. The CPG or supporting documents should provide a timeframe for review and updating AND should describe the process by which a decision is made to update and how the update will be conducted. These items do not need to be specific to the guideline.

© 2021 Burns KEA et al. *JAMA Network Open*.

eFigure. Identification of Clinical Practice Guidelines (CPGs)

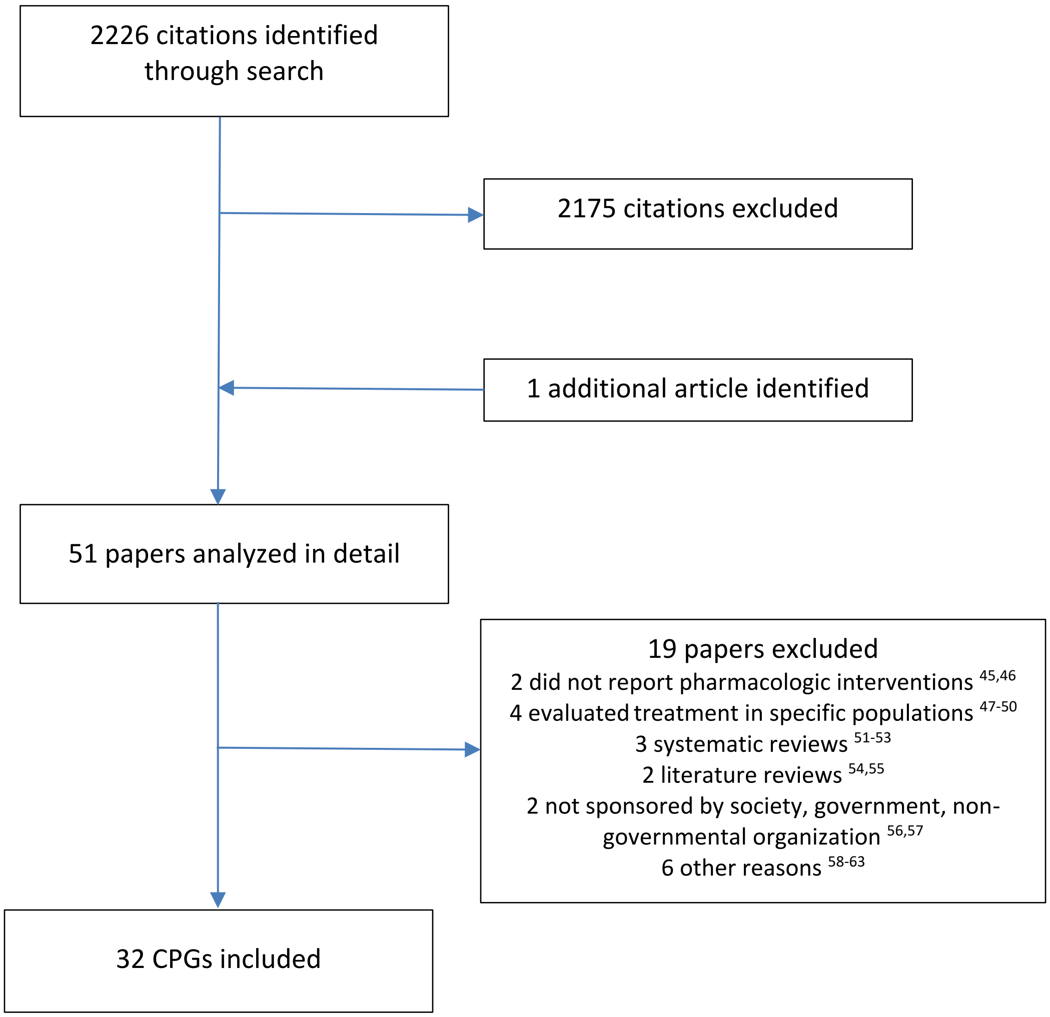

© 2021 Burns KEA et al. *JAMA Network Open*.

eTable 1. Characteristics of Included Clinical Practice Guidelines (CPGs)

**Feature**	**CPG (N= 32)**
Population addressed	
All hospitalized	29 (90.6%)
Ward only	0
ICU only	3 (9.4%)
CPG Scope	
International	15 (46.9%)
National	17 (53.1%)
State or province	0
CPG Sponsor	
Professional society	25 (78.1%)
Government agency	3 (9.4%)
Non-government/not-for-profit agency	4 (12.5%)
CPG Funding	
Yes	7 (21.9%)
No	12 (37.5%)
Uncertain	13 (40.6%)
Number of WHO regions guidelines emanated from	
1	20 (62.5%)
2	4 (12.5%)
3	2 (6.3%)
4	2 (6.3%)
5	2 (6.3%)
6	1 (3.1%)
Uncertain	1 (3.1%)
Patient and public perceptions sought (Yes)	2 (6.3%)

© 2021 Burns KEA et al. *JAMA Network Open*.

eTable 2. Summary of NEATS Scores for the Included Clinical Practice Guidelines (CPGs)

CPG Phase	CPG Design and Conduct					CPG Systematic Review of the Evidence			CPG Recommendations				Statement	CPG Completion	
NEATS Domain	Disclosure of CPG Funding Source	Disclosure of Financial COIs*	Multidisciplinary CPG Panel	Methodologist Included on CPG Panel	Patient and Public Perspectives	Search Strategy	Study Selection	Evidence Synthesis	Rating Quality or Strength of Evidence	Benefits and Harms	Evidence Summary	Rating Strength	Specific and Unambiguous Statement	External Review	Update
Yes	18		20	5											
No	14		12	26											
Unknown			0	1											
Score 1 or 2		12			30	26	26	25	22	18	20	22	8	28	30
Score 3		8			0	0	1	2	4	8	6	5	10	1	0
Score 4 or 5		12			2	6	5	5	6	6	6	5	14	3	2

Legend:
CPG = clinical practice guideline, COI = conflict of interest
* Refers to disclosure and management of financial conflicts of interest

© 2021 Burns KEA et al. *JAMA Network Open*.
