# Supplementary material for: Adherence of Clinical Practice Guidelines for Pharmacologic Treatments of Hospitalized Patients With COVID-19 to Trustworthy Standards: A Systematic Review
Source: JAMA Netw Open. 2021 Dec 10;4(12):e2136263. doi: 10.1001/jamanetworkopen.2021.36263 (PMC8665373; doi:10.1001/jamanetworkopen.2021.36263)
Supplement: Supplement 2. — Nonauthor Collaborators. The Academy of Critical Care: Development, Evaluation, and Methodology (ACCADEMY) [file jamanetwopen-e2136263-s002.pdf]

Supplemental Online Content: Nonauthor Collaborators

*Indicates required information. Only first name, last name, and suffix will appear in PubMed.

*Group Name(s): Academy of Critical Care: Development, Evaluation, and Methodology (ACCADEMY)							
***First Name and Middle Initial(s)**	***Last Name**	***Suffix (eg, Jr, III)**	Academic Degrees	Institution	Location (city, state/province, country)	Role or Contribution, eg, chair, principal investigator	Group (if more than 1 Group listed in the byline) and/or Subgroup (eg, Steering Committee)
David	Lightfoot		MISt, PhD	Unityhealth Toronto	Toronto, Ontario Canada	librarian, assisted with searches	none of the above
Karen	Choong		MD, MSc	McMaster Children's Hospital	Hamilton, Canada	ACCADEMY member	ACCADEMY
Andrea	Lucas		MSc candidate	Alberta Hospitals	Alberta, Canada	ACCADEMY member	ACCADEMY
Joanna	Dionne		PhD candidate	McMaster University	Hamilton, Canada	ACCADEMY member	ACCADEMY
Colungo	Lozano		MD, MSc	Hospital in Mexico	Mexico City, Mexico	ACCADEMY member	ACCADEMY
Erick	Duan		MD, MSc	Niagara Health Sciences Centre	St.Catherines, Ontario	ACCADEMY member	ACCADEMY
Emilie	Belley-Cote		MD, PhD	Sherbrooke University	Sherbrooke, Qc	ACCADEMY member	ACCADEMY
Melissa	Parker		MD, PhD	McMaster Children's Hospital	Hamilton, Canada	ACCADEMY member	ACCADEMY
John	Basmaji		MD, MSc	London Health Sciences Centre	London, Canaada	ACCADEMY member	ACCADEMY
Vincent	Lau		MD, MSc	London Health Sciences Centre	London, Canaada	ACCADEMY member	ACCADEMY
Samantha	Arora		MD, MSc	Thunder Bay Health Sciences Centre	Thunder Bay, Ontario	ACCADEMY member	ACCADEMY
